# Changes in leisure-time physical activity and sedentary behaviour at retirement: a prospective study in middle-aged French subjects

**DOI:** 10.1186/1479-5868-7-14

**Published:** 2010-02-04

**Authors:** Mathilde Touvier, Sandrine Bertrais, Hélène Charreire, Anne-Claire Vergnaud, Serge Hercberg, Jean-Michel Oppert

**Affiliations:** 1 UREN (Unité de Recherche en Epidémiologie Nutritionnelle), U557 Inserm, U1125 Inra, Cnam; Paris 13, CRNH IdF, F-93017 Bobigny, France; 2 Département de Santé Publique, Hôpital Avicenne, F-93017 Bobigny, France; 3 Service de Nutrition, Hôpital Pitié-Salpêtrière (AP-HP), Université Pierre et Marie Curie-Paris 6, CRNH-IdF, F-75013 Paris, France

## Abstract

**Background:**

Longitudinal studies on physical activity patterns around retirement age are scarce and provide divergent findings. Little is known about changes in sedentary behaviour in this context. Our aim was to investigate relationships between retirement and 3-year changes in leisure-time physical activity (LTPA) patterns and sedentary behaviour in middle-aged French adults.

**Methods:**

Past-year LTPA and sedentary behaviour (watching television) were assessed in 1998 and 2001 using the Modifiable Activity Questionnaire on participants in the SU.VI.MAX (Supplementation with Antioxidants and Minerals) study. A total of 698 men and 691 women aged 45-64 were included in this analysis. Comparisons were made between subjects who had retired between 1998 and 2001 and those who continued to work, using the Chi-square test, Student t-test, Wilcoxon rank test or covariance analysis where appropriate.

**Results:**

20.1% of men and 15.6% of women retired during follow-up. The baseline LTPA level was similar between subjects who retired during follow-up and those who continued to work. Mean LTPA increased by about 2 h/week in men and women who had retired, whereas no change was observed in employed persons. The positive change in LTPA following retirement was mainly related to an increase in activities of moderate intensity, such as walking. Retirement did not modify the ranking of the most frequently performed LTPAs, but the number of participants and the duration increased through retirement. In men, the increase in time spent watching TV was more than twice as high in retirees as in workers (+40.5 vs. +15.0 min/day, P < 0.0001). The same tendency was observed among women, but was borderline non-significant (+33.5 vs. +19.9 min/day, P = 0.05). In women, retirees who increased their walking duration by 2 h/week or more also decreased time spent watching TV by 11.5 min/day.

**Conclusions:**

Retirement was associated with both an increase in LTPAs and in time spent watching TV, suggesting that retirement is an important period not only for promoting physical activity, but also for limiting sedentary behaviour.

## Background

Physical activity (PA) is recognised to be a major protective factor against the development of several chronic diseases associated with ageing, such as type 2 diabetes, cardiovascular disease, weight gain and some cancers, as well as cognitive decline [1-3]. In older people, PA may also help to decrease falls and disability, and improve independence [4-6]. In addition to PA, there is increasing interest in the relationships between sedentary behaviour and health outcomes [7,8]. Sedentary behaviour refers to activities that do not substantially increase energy expenditure above the resting level; they include sleeping, sitting, lying down and watching television, along with other forms of screen-based entertainment [9]. Sedentary behaviour, typically assessed by time spent viewing television (TV), along with low PA levels, represent complementary aspects of human movement and are independent risk factors for major chronic diseases [2,10].

Retirement is a major transition in the life course and concerns a growing segment of the population. The number of persons who reach retirement age is rapidly increasing in developed countries. According to the World Health Organisation, in the year 2000, 600 million people were aged 60 or above throughout the world; this figure will reach 1.2 billion by 2025 and 2 billion by 2050 [11]. Retired persons generally have more time for leisure activities, potentially including leisure-time physical activity (LTPA, including structured activities such as sports, and unstructured activities such as walking for pleasure). Retired individuals may also have more time to spend on sedentary occupations such as TV viewing. However, the manner in which retirement affects PA and sedentary behaviour is poorly understood.

Cross-sectional surveys from the US and Europe suggested that PA patterns change at retirement [12-15]; however, few longitudinal data exist which describe these changes in the same individuals [16-20]. Discrepancies exist in the findings of these studies; one study found no relationship between retirement and sports activities or non-sports LTPA [18], while others observed that retirement was associated with an increase in sports activities and exercise [17] or increased overall PA [19,20]. Except for one study [17], detailed information on changes in specific leisure-time activities is lacking. Moreover, to our knowledge, only one study examined the effect of retirement on sedentary behaviour [17]; it concluded that retirement was associated with increased TV viewing. However, whether or not LTPA and sedentary behaviour changes are interrelated remains unknown.

The objective of the present study, which used a longitudinal design in a sample of middle-aged French adults, was to investigate the relationship between retirement and 3-year changes in LTPA and sedentary behaviour, as assessed by time spent watching TV.

## Methods

### Subjects

We used data from the "Supplémentation en VItamines Minéraux et AntioXydants" (SU.VI.MAX) study. The design, methods and rationale of this study have been described elsewhere [21]. The SU.VI.MAX study was initially designed as a randomised, double-blind placebo-controlled primary prevention trial to test the efficacy of daily supplementation with antioxidant vitamins and minerals at nutritional doses in reducing the incidence of ischaemic heart disease, cancers and overall mortality [22]. Volunteer subjects, not selected for specific risk factors, were included in 1994-1995 for a planned follow-up of 8 years (men: 45-60 y, women: 35-60 y). Each subject underwent a yearly visit consisting of alternate years of biological sampling or a clinical examination. This study was conducted according to guidelines laid down in the Declaration of Helsinki, and all procedures involving human subjects were approved by the Ethical Committee for Studies with Human Subjects at Paris-Cochin Hospital (CCPPRB N°706) and the Commission Nationale Informatique et Liberté (CNIL N°334641). Written informed consent was obtained from all subjects.

### Physical activity and sedentary behaviour

PA and sedentary behaviour were assessed using a French self-administered version of the Modifiable Activity Questionnaire (MAQ) [23] sent out in 1998 and 2001 to the entire cohort. The MAQ, developed by Kriska et al. [24], was the instrument used in the Diabetes Prevention Programme [25]. It assesses past-12-month PA during leisure time and at work. PA assessment using the MAQ has been validated against energy expenditure measurements using the double-labelled water technique, and the test-retest properties of the questionnaire have been shown [26]. The questionnaire has been described in detail elsewhere [23,24,26-28]. Briefly, subjects were asked to report all LTPAs performed at least 10 times for 10 min per session over the past 12 months. Detailed information was collected concerning the type of leisure activity (walking, cycling, swimming, jogging, gardening, etc.). The frequency and duration of each activity was reported. After multiplying the number of hours per week of each PA by its estimated metabolic cost (in MET [29]), an energy-expenditure indicator was obtained, expressed in MET-h per week. Leisure time activities were classified into three categories of intensity: low (<3 METs), moderate (3-6 METs) and vigorous (>6 METs). Assessment of occupational PA was based on the number of hours during which an individual participated in physically demanding activities during an average work day, for each job held over the past year. The number of hours in each of three categories of occupational PA (low, moderate, and vigorous) was multiplied by an average group MET value (2, 4 and 7 METs, respectively) and then summed up, resulting in a final occupational activity estimate expressed in MET-h/week. Subjects who declared only low-intensity occupational physical activities were considered to have a "less physically demanding" job, while others (who declared at least one moderate and/or vigorous occupational activity) were considered to have a "more physically demanding" job.

Subjects were considered to meet overall PA recommendations if their overall PA was ≥ 60 min per week of vigorous activities with at least 20 min per session or ≥ 150 min per week of moderate activities [3,30,31]. The questionnaire also assessed time spent per day watching TV (min/day) as an indicator of sedentary behaviour.

### Sociodemographic characteristics

Employment status (currently working/retired) was assessed during the 1998 and 2001 follow-up visits. Gender, date of birth and education level were assessed at enrollment using a self-administered questionnaire. Level of education was coded into three categories according to highest certification obtained (primary school, high school, university or equivalent). The type of area of residence (urban area, periurban zone or rural municipality) was determined according to each subject's zip code in January 1998, according to the definition of the French National Institute of Statistics and Economics Studies [32], as previously described [27]. Current smoking status was assessed in September 1998 by a specific questionnaire sent to the entire cohort. Height, weight and waist circumference were measured during the 1998 and 2001 follow-up visits. BMI was calculated as body weight (in kilograms) divided by height squared (kg/m^2^). Obesity was defined by a BMI ≥30 kg/m^2 ^[33].

### Statistical analyses

Among the 13,017 subjects initially included in the SU.VI.MAX study, we focused the present analyses on subjects aged 45-64 years old (to have a similar age range in both genders) (7,450 subjects available), with available data from PA questionnaires both in 1998 and 2001 (2,881 remaining subjects), with available data on working status both in 1998 and 2001 and who were working in 1998 (1,389 remaining subjects for analysis, 698 men and 691 women). We verified that, in these subjects, none had been confined to bed for more than 1 month during the 12-month period covered by each PA questionnaire. Compared to those subjects not included and aged 45-64 years old (n = 6,061), our study population comprised more men (50.3 vs. 44.7%), more subjects with a university education level (49.7 vs. 35.7%), and fewer obese subjects (6.5 vs. 9.2%); they were also slightly younger (52.7 vs. 54.4 y) and had a lower waist circumference (82.2 vs. 83.2 cm) (P < 0.05).

For each subject, we evaluated the evolution between 1998 and 2001 of LTPA (duration in h/week and score in MET-h/week, overall and by category of LTPA intensity) and of time spent watching TV. Comparisons of baseline characteristics were performed between subjects who retired between 1998 and 2001 and those who continued to work using the Chi-square test, Student t-test or Wilcoxon rank test where appropriate. Three-year changes (1998-2001) in LTPA and time spent watching TV between the 2 groups of individuals were compared by covariance analysis, with adjustment for age, education level and baseline value of the corresponding variable. These comparisons were conducted overall and stratified according to adherence to PA recommendations and the physical demands of work (the extent of physical effort required, i.e., more demanding or less demanding) in 1998. We also established a ranking of the most frequently performed LTPAs in 1998 and 2001 among subjects who retired during follow-up, in order to analyse potential gender-specific or retirement-related patterns. Finally, we assessed changes in TV viewing among retirees according to the evolution of time spent walking between 1998 and 2001. Analyses were stratified on gender. For all analyses, the significance level was two-sided and set at 0.05. All statistical analyses were performed using SAS software (version 9.1, SAS Institute Inc, Cary, NC, USA).

## Results

### Baseline characteristics of the study population

20.1% of men (n = 140) and 15.6% of women (n = 108) retired during follow-up (Table 1). These subjects were older than those who had still been working in 2001. In men, retirees had a lower education level. No significant difference was found at baseline for type of resident location, smoking status, BMI, prevalence of obesity or waist circumference. The baseline LTPA level was similar between subjects who retired and those who continued to work (Table 2). Baseline time spent watching TV was higher (of 11.3 min/day in men and 10.0 min/day in women) in subjects who retired during follow-up, but the difference was significant only in men. There was no difference at baseline in the proportion of subjects meeting overall PA recommendations between those who continued to work after follow-up and those who retired (67.6% vs. 68.6%, P = 0.8 in men, and 60.7% vs. 57.4%, P = 0.5 in women, data not tabulated).

**Table 1:** Sociodemographic and anthropometric characteristics at baseline in 1998, according to working status in 2001

	Men			Women		
		
	Working (n = 558)	Retired (n = 140)	P	Working (n = 583)	Retired (n = 108)	P
Age (years)^1^	52.3 ± 3.0	57.1 ± 3.2	< 0.0001	51.2 ± 3.1	56.3 ± 3.7	< 0.0001
Education level			0.03			0.8
Primary school	15.7%	19.3%		11.8%	12.5%	
High school	29.6%	38.6%		40.5%	43.3%	
University or equivalent	54.7%	42.1%		47.7%	44.2%	
Type of resident location			0.6			0.9
Urban pole	65.0%	69.3%		64.5%	66.7%	
Periurban zone	17.4%	15.0%		18.3%	17.6%	
Rural municipality	17.6%	15.7%		17.2%	15.7%	
Current smokers	14.5%	11.4%	0.3	11.5%	7.4%	0.2
BMI (kg/m^2^)^1^	25.2 ± 3.2	25.4 ± 3.2	0.5	23.3 ± 3.8	23.8 ± 3.0	0.2
≥ 30 kg/m^2^	7.2%	7.2%	1.0	5.8%	5.3%	0.8
Waist circumference (cm) ^1^	89.2 ± 9.3	90.1 ± 10.0	0.4	74.8 ± 9.1	76.7 ± 8.7	0.06

^1 ^Mean ± standard deviation, P-value from Student t-test.

**Table 2:** Baseline and 3-year changes in leisure-time physical activities (LTPAs) and time spent watching TV according to working status in 2001

	Men			Women		
		
	Working (n = 558)	Retired (n = 140)	P	Working (n = 583)	Retired (n = 108)	P
Baseline^1^						
						
LTPA						
Duration (h/week)	3.7 ± 3.9	3.9 ± 3.7	0.4	3.4 ± 3.6	3.9 ± 4.1	0.4
Score (MET-h/week)	19.0 ± 20.2	19.1 ± 19.8	0.8	15.4 ± 17.0	17.4 ± 18.8	0.6
Occupational PA						
Duration (h/week)	19.1 ± 12.0	18.5 ± 13.0	0.4	18.4 ± 13.2	15.9 ± 12.8	0.05
Score (MET-h/week)	59.9 ± 64.2	59.2 ± 72.8	0.4	50.5 ± 50.9	43.6 ± 49.9	0.04
Time spent watching TV						
Duration (min/day)	98.7 ± 58.1	110.0 ± 56.8	0.02	99.2 ± 57.8	109.2 ± 59.3	0.1
						
Changes^2 ^between 1998 and 2001						
						
Δ LTPA						
Duration (h/week)	-0.1 ± 0.2	2.1 ± 0.4	< 0.0001	-0.4 ± 0.1	1.8 ± 0.4	< 0.0001
Score (MET-h/week)	-0.5 ± 0.9	8.1 ± 1.9	0.0001	-1.9 ± 0.6	6.8 ± 1.6	< 0.0001
Δ LTPA by intensity (h/week)						
Low intensity (<3 METs)	-0.0 ± 0.0	0.1 ± 0.0	0.06	-0.0 ± 0.0	0.2 ± 0.1	0.005
Moderate intensity	0.0 ± 0.2	2.0 ± 0.4	< 0.0001	-0.3 ± 0.1	1.6 ± 0.3	< 0.0001
Vigorous intensity (>6 METs)	-0.1 ± 0.1	0.0 ± 0.2	0.4	-0.1 ± 0.0	0.1 ± 0.1	0.1
Δ Leisure-time walking duration (h/week)	-0.1 ± 0.1	0.8 ± 0.1	< 0.0001	-0.2 ± 0.1	1.2 ± 0.2	< 0.0001
Δ Time spent watching TV (min/day)	15.0 ± 2.3	40.5 ± 5.0	< 0.0001	19.9 ± 2.5	33.5 ± 6.3	0.05
Δ BMI (kg/m^2^)	0.4 ± 0.1	0.4 ± 0.1	1.0	0.6 ± 0.1	0.6 ± 0.2	0.9
Δ Waist circumference (cm)	1.1 ± 0.3	1.6 ± 0.6	0.4	1.9 ± 0.3	3.3 ± 0.8	0.1

^1 ^Mean ± standard deviation, P-value from Wilcoxon rank test.

^2 ^Mean ± standard error, P-value from ANOVA models, adjusted for age, education level and baseline value of the corresponding variable (i.e. baseline: LTPA duration, LTPA score, LTPA low, moderate or vigorous intensity, leisure walking duration, time spent watching TV, BMI, or waist circumference, respectively).

### Influence of retirement on leisure-time physical activity and sedentary behaviour

After adjustment for baseline LTPA level, age and education level, mean LTPA increased by about 2 h/week between 1998 and 2001 in men and women who had retired, whereas no change was observed in those who continued to work (Table 2). In both genders, retirement was associated with an increase in LTPA whether or not subjects initially attained recommended PA levels (P < 0.0001 and P = 0.01, respectively, data not tabulated). The increase of LTPA after retirement was also observed whether subjects had a more or a less physically demanding job at baseline, before retirement (P < 0.001 and P < 0.0001, respectively, data not tabulated). In both genders, the increase in LTPA at retirement was mainly explained by an increase in activities of moderate intensity, including leisure-time walking (Table 2).

Among subjects who retired during follow-up, the five most frequent LTPAs were walking, gardening, cycling, swimming and running (for men), and walking, gardening, gymnastics, swimming and hiking (for women) (Table 3). Retirement did not modify this gender-specific ranking, but the number of participants in the main activities (notably walking) as well as mean duration per week increased at retirement.

**Table 3:** Ranking of the five most frequently performed leisure-time physical activities in 1998 and in 2001 among subjects who retired during follow-up

Men (n = 140)			Women (n = 108)		
	
Activity	% of participants	Mean duration (h/week) among participants ± std	Activity	% of participants	Mean duration (h/week) among participants ± std
1998			1998		
					
Walking	60.0	1.3 ± 1.1	Walking	67.6	1.7 ± 1.7
Gardening	51.4	2.6 ± 2.8	Gardening	48.1	2.2 ± 2.4
Cycling	22.1	1.6 ± 1.5	Gymnastics	27.8	1.0 ± 0.6
Swimming	19.3	0.4 ± 0.3	Swimming	26.9	0.5 ± 0.5
Jogging/running	15.7	1.9 ± 2.4	Hiking	14.8	2.4 ± 1.4
					
					
2001			2001		
					
Walking	70.7	2.4 ± 2.5	Walking	72.2	2.8 ± 4.7
Gardening	52.9	4.0 ± 4.6	Gardening	49.1	2.5 ± 2.6
Cycling	21.4	2.0 ± 2.1	Gymnastics	29.6	1.3 ± 1.0
Swimming	17.1	0.9 ± 1.5	Swimming	25.9	0.5 ± 0.3
Jogging/running	14.3	1.2 ± 1.0	Hiking	13.9	2.0 ± 1.4

Among retirees, after adjustment for age, education level and score at baseline, the overall PA score (i.e. occupational PA + LTPA in 1998 and LTPA in 2001; occupational PA being null after retirement by definition) decreased by 50.7 MET-h/week (SE = 2.3) in men and 41.8 MET-h/week (SE = 1.9) in women between 1998 and 2001, with no statistically significant difference across physical demands at work in 1998 (P = 0.4 in men and P = 0.6 in women, data not tabulated).

**Figure 1:**
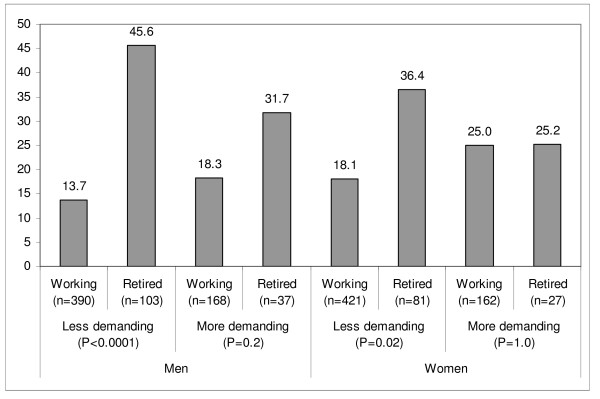
**Three-year changes in time spent watching TV (min/day) according to initial physical demands at work^1^**. ^1^Adjusted for age, education level and baseline time spent watching TV.

**Figure 2:**
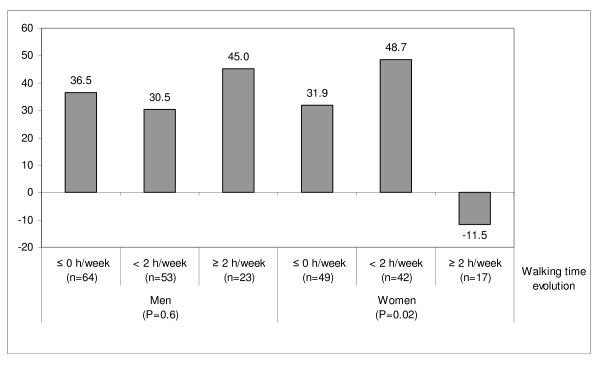
**Three-year changes in time spent watching TV (min/day) among subjects who retired during follow-up, according to evolution of time spent walking between 1998 and 2001^1^**. ^1^Adjusted for age, education level and baseline time spent watching TV.

Retirement was associated with an increase in time spent watching TV, which increased twofold in retired men compared to working men (Table 2). When stratifying the analyses according to physical demands at work in 1998, we observed that the increase in TV viewing at retirement was statistically significant only in men and women who had previously performed a less physically demanding job (Figure 1). Since walking was the most frequent LTPA and was the main contributor to the retirement-related LTPA increase, we assessed changes in TV viewing among retirees according to the evolution in time spent walking (Figure 2). Changes in TV viewing differed according to category of changes in walking habits only in women; those who increased their duration of walking by 2 h/week or more concomitantly decreased their time spent watching TV by an average of 11.5 min/day.

## Discussion

Our study assessed the influence of retirement on LTPA and sedentary behaviour in French subjects. In both genders, retirement was associated with an increase in LTPA, especially with increased duration of moderate LTPA and walking. Retirement did not modify the ranking of the most frequently performed LTPAs, but the number of participants and the duration increased at retirement. In subjects who retired from less physically demanding jobs, retirement was associated with an increase in time spent watching TV. In women retirees, a high increase in time spent walking was associated with a decrease in TV viewing.

In our study, the retirement-related increase in LTPA was substantial, as it represented about 2 h/week in both genders. This increase in LTPA is consistent with previous findings [17,19,20]. However, unlike previous studies that showed modifications only in population subgroups [16], we observed a positive impact of retirement on LTPA whether or not subjects initially met recommended PA levels and whatever the physical demands at work prior to retirement. An increase in LTPA of 2 h/week on average may be substantial in terms of potential health-related benefits. A recent trial examined in inactive overweight women the effect of exercising at 50%, 100%, and 150% of the currently recommended physical activity dose (150 min/week of moderate-intensity physical activity [1,2]) on cardiorespiratory fitness [34]. Subjects experienced a graded dose-response change in fitness across levels of exercise training, with statistically significant improvement observed even for the lowest intervention level [34]. Increases in cardiorespiratory fitness are associated with decreased mortality risk [1,2].

There are several potential explanations for an increase in LTPA with retirement, including reduced time-related barriers, long-term perspective on health, concern about health and independence and changes in social networks, support systems and daily routines [17,35]. However, in our study, the increase in LTPA associated with retirement did not compensate for the loss of occupational PA.

The effect of retirement assessed in our study may be a short-term effect, corresponding to what happened 1-to-3 years after retirement. Caution should be used when extrapolating these findings to the entire post-retirement period. Indeed, cross-sectional data suggested that at retirement, there existed a tendency in improving physical activity patterns, but this positive effect decreased with time after retirement, to disapeare during the final period of life [15]. In the GLOBE study [18], including a 13-year interval between baseline and final PA measures, no significant relationship was observed between retirement and LTPA. One can hypothesize that there may have been an increase in LTPA in the first years following retirement, but this increase was no longer visible 10-13 years after retirement.

Our study offered the opportunity of investigating in detail specific LTPAs performed in each gender and potential modifications of such LTPA patterns during retirement. One major finding was that retirement quantitatively modified the LTPA pattern (an increase in the number of participants and in the duration), but not qualitatively (there was no change in gender-specific ranking of the most frequent LTPAs). In our study, walking was the most frequently performed LTPA and was the main contributor to the retirement-related LTPA increase. In the ARIC study, walking was the most frequently cited activity by subjects who adopted a LTPA or a sports activity at retirement [17]. Walking is easily performed, requires no specific equipment, does not incur any substantial cost and is the basis for public health recommendations concerning PA [2].

In our study, the increase in LTPA associated with retirement occurred concurrently with an increase in time spent watching TV. Changes in time spent watching TV had been assessed in only one previous study on retirement, in which an increase was observed, in agreement with our results [17]. The relationship between PA and sedentary behaviour is complex; it has been pointed out that 'sedentary' is not simply the opposite of 'active' [9,10,36]. Several studies have reported that sedentary behaviour and PA are related to risk of major chronic disease independently of one another [27,37-39]. Some of our findings contrast with this notion of independence of PA and sedentary behaviour. Indeed, we found that subjects who had held a less physically demanding profession were more inclined to adopt sedentary behaviour during their leisure time once retired, independently of age or education level. This suggests that this group should be targeted for measures aimed at limiting increases in sedentary behaviour at retirement. Moreover, overall, the retirement related 2 h/week increase in LTPA did not compensate for the 30-40 min/day increase in TV viewing. However, in stratified analyses, only women who declared the highest increase in walking duration after retirement simultaneously decreased TV viewing. This suggests that certain groups of population exist for whom the benefit of retirement is enhanced, since these subjects compensate for the decrease in sedentary occupations by increasing their LTPAs.

In contrast to our highly-detailed PA questionnaire, our proxy for sedentary behaviour was limited to time spent watching TV, which may have led to an underestimation of time devoted to sedentary occupations. To better assess attributes of a sedentary lifestyle, an evaluation of time spent sitting down while traveling and at work, along with other screen-based sedentary occupations such as computer use during leisure time, would be of interest. Indeed, sedentary lifestyle represents a complex set of behaviours which may each have separate effects on health outcome [28,40] and may be modified differentially by retirement. It should be noted, however, that in women, TV viewing has been suggested as a robust marker of overall sedentary lifestyle [41].

Several limitations to our study should be pointed out. First, despite a mean retirement age in our population (57-60 y) consistent with retirement age in France in the early 2000's (55 y for some professions such as teachers, and 60 years for the majority of other professions, mean age: 58.1 y [42]), our subjects were volunteers participating in a nutritional intervention study [22] who generally had a higher education level and occupational status, along with a healthier lifestyle than the general population [21]. Thus, our study sample cannot be considered as nationally representative of individuals aged 45-64, and caution is needed when extrapolating these findings to the national level. Our analyses focused on a part of the SU.VI.MAX cohort; however, sociodemographic differences observed between those subjects who were and those who were not included in the analyses were unlikely to have caused additional bias in the studied associations. Second, measurements of LTPA and TV viewing were derived from self-reporting, which may be a source of potential misclassification bias (especially due to over-reporting of PA [43]). However, there is no reason to expect that misclassification would differ according to retirement status. Third, our study did not explore the reasons for retirement, which may have differential effects on PA and other types of health behaviour. A possible selection bias could have occurred if participants in poor health had retired earlier for that reason, and if poor health rather than retirement per se negatively influenced PA. However, in our study, this type of bias would not explain the observed increase in LTPA and would have minimised the true effect. Moreover, a previous study showed that tobacco use and alcohol intake, but not PA, were differentially affected by voluntary or involuntary retirement [19]. Validity of using the MAQ in older subjects and in a longitudinal design may be discussed. For reliability, in the first description of the MAQ by Kriska et al. [24], 1-3 week test-retest correlations for past-year LTPA were found of about the same magnitude in the older compared to the younger adult subjects (37-59 y, rho = 0.88 and 21-36 y, rho = 0.92, respectively). For validity (i.e. comparison against a gold standard method), we are not aware of study data that have assessed the performance of the MAQ according to age groups. Regarding the appropriateness of using the MAQ in a longitudinal design, repeated use of this questionnaire allowed to detect annual evolution of PA during follow-up (median: 2.8 y) in the Diabetes Prevention Program [25]. In a subsample of DPP participants (n = 274, 50.6 ± 11.3 y), LTPA (MET-h/week) measured by the MAQ was correlated with self-reported minutes of physical activity at both 1 year (r = 0.41, P < 0.0001) and 2 years (r = 0.51, P < 0.0001) [44].

One of the strengths of our study lay in its prospective design and the use of a detailed PA questionnaire, which specified the types of LTPA performed during the preceding year and the duration of each reported activity. In addition, to our knowledge, only one previous study analysed the influence of retirement on both PA and sedentary behaviour [17]. Our data enabled adjustment for confounding factors and considered individual heterogeneity by stratified analyses. Moreover, in previous studies [12,17,18], definitions of work and retirement were not mutually exclusive, since some subjects were considered as retired, but were still working part-time or full-time, and retirement status was reversible. In our study, the definition of retirement was less ambiguous: we considered retired persons to be subjects who had definitively stopped all professional activity. Indeed, in France, in 1998-2001, people were considered "retired" and started to receive their retirement allocations only when they stopped any professional activity. Thus, a new profession would not have been allowed. As for potential volunteer implication in associations or charities, corresponding physical activity was counted as LTPA.

## Conclusions

Our study provides important information about retirement-related changes in physical activity and sedentary behaviour. Few data exist on this topic, despite the increasing number of ageing individuals concerned [11] and the major health consequences related to insufficient physical activity and excessive sedentary behaviour [1-3]. Our results suggest that retirement is a key period of change in PA and sedentary behaviour. The substantial 2 h/week increase in LTPA through retirement observed in our study did not compensate for loss of occupational PA. This emphasises the need to reinforce public health measures aimed at increasing regular PA and decreasing sedentary behaviour during the critical retirement period, as well as during other lifetime transition periods.

## Competing interests

The authors declare that they have no competing interests.

## Authors' contributions

MT performed statistical analysis, interpreted results and drafted the manuscript. SB contributed to designing the study, performing analyses and interpreting results. HC and ACV contributed to interpreting results and editing the manuscript. SH is coordinator of the SU.VI.MAX study and contributed to interpreting results and editing the manuscript. JMO supervised the design, analysis and interpretation of data as well as writing of the manuscript. All authors critically reviewed the manuscript and approved the final version.
